# Granulomatous reaction after facial filler during metastatic melanoma treatment with immunotherapy: a case report^☆^

**DOI:** 10.1016/j.abd.2024.01.007

**Published:** 2024-10-28

**Authors:** Luana Pizarro Meneghello, Diéssica Gisele Schulz, Verônica Hamann Aita, Marcio Freitas Valle de Lemos Weber

**Affiliations:** a Dermatologist, Universidade Franciscana, Santa Maria, RS, Brazil; b Department of Dermatology, Hospital Universitário de Brasília, Brasília, DF, Brazil; c Department of Dermatologia, Universidade Federal de Ciências da Saúde de Porto Alegre, Porto Alegre, RS, Brazil; d Radiologist, Private Practice, Santa Maria, RS, Brazil

Dear Editor,

Increase in life expectancy favors the search for aesthetic interventions that aim to eliminate the undesirable signs of aging. Dermal fillers are commonly used in dermatology practice to overcome changes resulting from the skin aging process, but foreign body reactions can occur, especially in the presence of risk factors. This case report describes a case of granulomatous reaction in areas of a previously performed aesthetic procedure, in a 79-year-old woman with malignant melanoma undergoing immunotherapy treatment, which is still rarely reported in the dermatological literature.

A 79-year-old woman, with a history of nodular melanoma excision on the right 5th toe (T3b), progressed with in-transit metastases two years after the diagnosis. Linearly distributed nodules were observed bilaterally in the periocular and in the glabella region 16 weeks after starting pembrolizumab (Fig. 1). The patient reported a history of injectable procedures for aesthetic purposes two years before the melanoma diagnosis. She did not remember which injectable product was used.

**Fig. 1:**
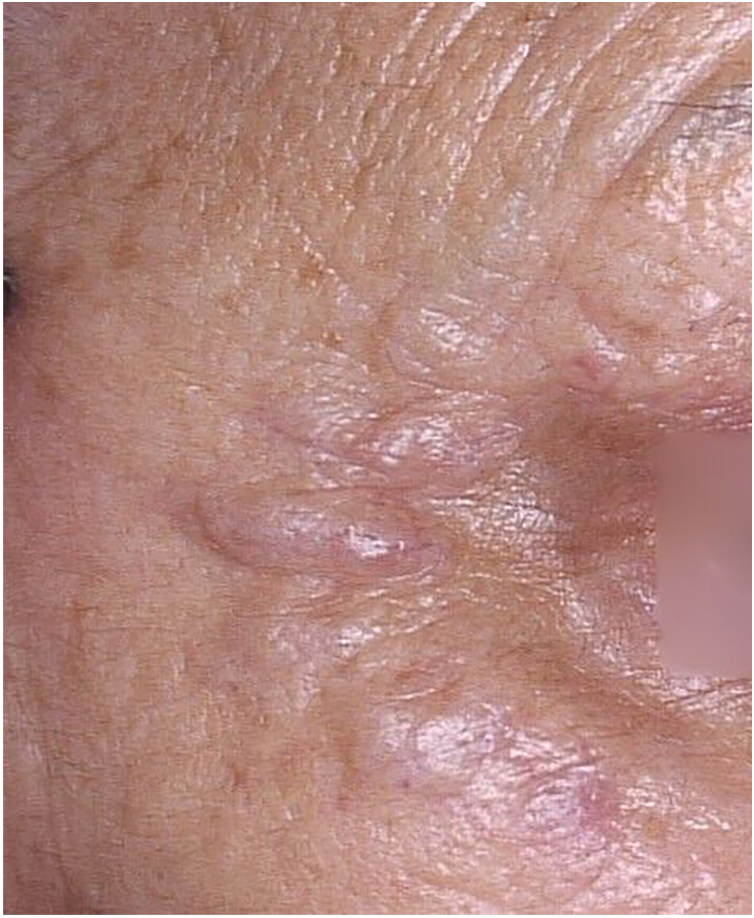
Nodules in the periocular region.

Dermatological ultrasound showed bilateral subcutaneous thickening in the periorbital region, especially in the lateral aspects; a similar process was seen in the frontal and dorsal regions of the base of the nose (Fig. 2A). In these regions, some areas with a grossly nodular, hyperechoic appearance were observed, with posterior acoustic shadow suggesting the presence of calcifications, the largest measuring 1.2 cm in the nasal region; 0.6 cm in the lateral region of the right orbit and approximately 0.9 cm in the left periorbital region; infiltration and reduced echogenicity in the hypodermis of the frontal region and mild infiltration in the hypodermis involving the periorbital region bilaterally. There was also a small increase in vascularization.

**Fig. 2:**
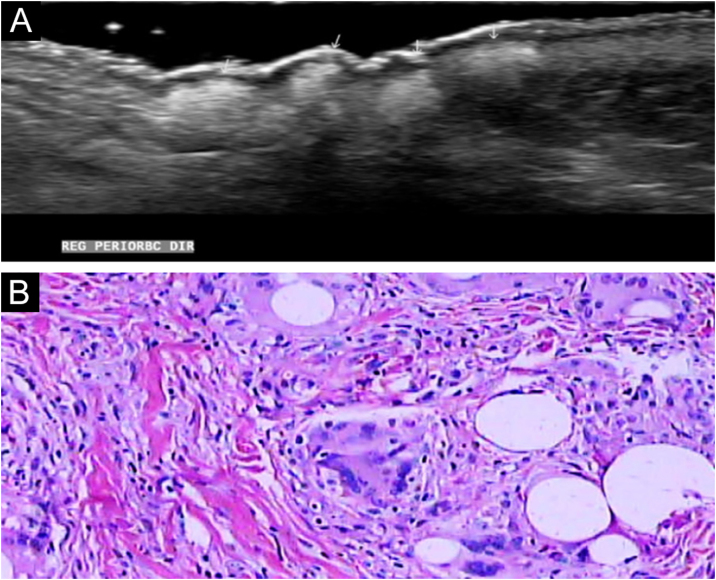
(A) Ultrasonography showing hyperechoic areas coarsely nodular; (B) Histopathology showing a granulomatous inflammatory process, with giant cells in the dermis phagocytizing empty and round vacuoles of different sizes, without evidence of necrosis (Hematoxylin & eosin, ×400).

Histopathology showed an extensive granulomatous inflammatory process, with giant cells in the dermis phagocytizing empty and round vacuoles of different sizes; without evidence of necrosis or presence of neoplastic cells (Fig. 2B).

Twenty-four weeks after starting immunotherapy, she also developed a lichenoid reaction on the upper limbs. It was decided, together with the patient, not to perform a procedure to remove the granulomas or use systemic corticosteroids due to the current immunotherapy, maintaining the follow-up.

Facial enhancement with dermal fillers for cosmetic purposes has been increasing in recent years and the procedures are not free of complications. Earlier complications after the procedure such as infection, hypersensitivity reaction, skin discoloration, vascular occlusion and irregularity may occur. After months or years, a granulomatous foreign body reaction, migration of material and scarring may also occur.

Some experts advocate the clinical diagnosis of late inflammatory reactions, in the presence of firm nodules that appear months or years after an injectable procedure. Others consider ultrasound imaging to be the reference examination due to the capacity to specify the location of late-onset nodules, as well as demonstration of the density of the filler. Histopathology and cultures can help in the differential diagnosis to rule out infection. There is no consensus on the best management of late inflammatory reactions.^1^ Therefore, it was decided not to send material to be cultured in this case.

In the setting of metastatic melanoma, better overall survival can be achieved with immunotherapy. One example is anti-PD-1 drugs (pembrolizumab or nivolumab). However, increased response and survival rates are also associated with immune system-related adverse events. Around 30%–40% of patients treated with anti-PD1 drugs may present diverse cutaneous manifestations.^2^ Some cases of foreign body-type reactions have already been described in patients undergoing fillings while using biological therapy for different diseases.^3^, ^4^

Foreign body granulomatous inflammation involves five phases: recognition of inflammation, protein adsorption, macrophage adhesion, macrophage fusion, and crosstalk between inflammatory cells. Granulomas consist of multinucleated giant cells, T-lymphocytes, histiocytes and fibroblasts. The histopathological characteristics of foreign-body granulomas vary depending on the filler used.^5^

Hypersensitivity reactions to fillers and other complications of advanced aesthetic procedures in patients undergoing immunotherapy, such as the case described in the present report, are challenging situations and still little reported in the literature. With the increasing prevalence of cosmetic injections, dermatologists should consider a granulomatous reaction related to the aesthetic procedure in patients who develop any skin alterations during treatment with immunobiologicals.

## Financial support

None declared.

## Authors' contributions

Luana Pizarro Meneghello: Effective participation in research orientation; intellectual participation in the propaedeutic and/or therapeutic conduct of the studied cases; critical review of the manuscript; approval of the final version of the manuscript.

Diéssica Gisele Schulz: Drafting and editing of the manuscript; critical review of the literature; approval of the final version of the manuscript.

Verônica Hamann Aita: Drafting and editing of the manuscript; critical review of the literature; approval of the final version of the manuscript.

Marcio Freitas Valle de Lemos Weber: Effective participation in research orientation; approval of the final version of the manuscript.

## Conflicts of interest

None declared.
